# Nanofluid to Nanocomposite Film: Chitosan and Cellulose-Based Edible Packaging

**DOI:** 10.3390/nano10040660

**Published:** 2020-04-02

**Authors:** Mekro Permana Pinem, Endarto Yudo Wardhono, Frederic Nadaud, Danièle Clausse, Khashayar Saleh, Erwann Guénin

**Affiliations:** 1 Chemical Engineering Department, University of Sultan Ageng Tirtayasa, Jl Jendral Sudirman km 3, Cilegon 42435, Banten, Indonesia; mekro-permana.pinem@utc.fr; 2 Integrated Transformations of Renewable Matter Laboratory (EA TIMR 4297 UTC-ESCOM), Sorbonne Universités, Université de Technologie de Compiègne, rue du Dr Schweitzer, 60200 Compiègne, France; daniele.clausse@utc.fr (D.C.); khashayar.saleh@utc.fr (K.S.); 3 Service d’Analyse Physico-Chimique (SAPC), Sorbonne Universités, Université de Technologie de Compiègne, rue du Dr Schweitzer, 60200 Compiègne, France; frederic.nadaud@utc.fr

**Keywords:** chitosan, cellulose, nanofluid, nanocomposite film

## Abstract

Chitosan (CH)-based materials are compatible to form biocomposite film for food packaging applications. In order to enhance water resistance and mechanical properties, cellulose can be introduced to the chitosan-based film. In this work, we evaluate the morphology and water resistance of films prepared from chitosan and cellulose in their nanoscale form and study the phenomena underlying the film formation. Nanofluid properties are shown to be dependent on the particle form and drive the morphology of the prepared film. Film thickness and water resistance (in vapor or liquid phase) are clearly enhanced by the adjunction of nanocrystalline cellulose.

## 1. Introduction

Chitosan is an amino polysaccharide derived from alkaline hydrolysis of chitin, an abundant polymer occurring in nature that is found in the exoskeletons of crustaceans and the cell wall of fungi [1]. Chitosan offers many advantages with great potential industrial applications due to its biodegradability, biocompatibility, antibacterial activity, nontoxicity, and versatile chemical and physical properties [2,3]. Chitosan also possesses an excellent film-forming ability that can be employed as a green alternative to food packaging films. It is considered to be a good candidate to replace petroleum-based polymers, to reduce waste inputs to the environment and to decrease health hazards due to the removal of toxic additives from nonbiodegradable plastics such as polyethylene, polypropylene, and polystyrene into the consumable products [4,5].

Fabrication of chitosan-based films for packaging applications has been widely studied [6,7]. However, as a forming film consisting of a single component, chitosan is still inadequate in practical use. Major limitations are low mechanical properties, especially in terms of ability to elongate, poor heat resistance, and weak moisture barrier ability compared to plastic films [8,9,10]. Moreover, it cannot be molded or even be heat-sealed like thermoplastic polymers. The physicochemical properties of chitosan depend on the molecular weight and degree of deacetylation (DD) that affects their functionalities [11]. An alternative method to improve its properties to an acceptable level is obtained by incorporating reinforcement materials to form nanocomposites. Chitosan is a highly compatible material, thus it is generally useful when blended with nanomaterials. Therefore, it can be used to produce biobased nanocomposites via mixing with Cellulose Nanocrystals (CNC) [12,13].

CNC are needlelike cellulose crystals of 10–20 nm in width and several hundred nanometers in length that are produced from various fiber sources (e.g., bleached wood pulp, cotton, manila, tunicin, or bacteria) by removing the amorphous regions while keeping the crystalline regions through partial depolymerization [14,15,16]. CNC is highly crystalline, and it has a large aspect ratio and ability to form interconnected network structures through hydrogen bonding [17]. The major challenge associated with the nanofabrication of composite materials prepared with CNC is to obtain an appropriate percolation network [18], because CNC exhibit strong hydrogen bonding interactions between one another [19,20]. Large undesirable agglomerates can be created within the polymer, while at the same time the same interactions play an important role to produce the desirable filler network inside the matrix.

To maximize interfacial adhesion within the polymer, it is essential to disperse the CNC into the matrix polymer in order to maintain the filler/filler hydrogen bonding interactions [19]. Many strategies have been adopted, like the use of surfactants or using the chemical surface modification of the nanowhiskers [21,22,23,24]. This strategy is prohibited by the use of surfactants to coat the high specific area of the nanocrystals [25]. Polymer chain surface modification of the nanoparticles is an alternate way to prepare nanocomposites with CNC [19,26,27,28]. Mesquita et al. [17] prepared biobased nanocomposites via covalent linkage between chitosan and functionalized CNC.

In this work, biobased nanocomposites were obtained through the simple mixing between CNC and nanosized chitosan. The films were prepared by mixing in a liquid phase and subsequent evaporation. Several studies were carried out to observe the relation between the composition of the suspension in the liquid phase and the morphology of nanocomposite film and decipher the phenomena underlying film formation.

## 2. Materials and Methods

### 2.1. Materials

Chitosan from shrimp shell (low molecular weight grade, DD 75–85%) was purchased from Sigma-Aldrich, Saint-Quentin Fallavier, France. Glacial acetic acid, glycerol, and sodium tripolyphosphate (TPP) were obtained from Thermo Fisher, Scientific, Illkirch-Graffenstaden, France, and CNC were bought from CelluForce, QC, Canada. Demineralized water (conductivity of 0.06 mS cm^−1^) produced by a purification chain (Veolia, Paris, France) was used for all experiments. 

### 2.2. Preparation of Solutions

TPP solution (0.5% w/v) and CNC suspension (1% w/v) were prepared, respectively, by dissolving 0.5 g of granular TPP and 1 g of CNC powder in a beaker glass containing 100 mL water. Each solution was then stirred at 300 rpm using a magnetic stirrer at room temperature for 2 h, while 0.5% w/v of chitosan (CH) solution was prepared by dispersing 0.5 g of chitosan powder and 0.1 g glycerol in a beaker glass containing 100 mL of a buffer, which is 0.10 M in acetic acid, then stirred up to 500 rpm using a magnetic stirrer for 24 h. The resulting CH was then filtered using Whatman filter paper no. 1 to remove the impurities.

### 2.3. Preparation of Nanochitosan Particles

Nanochitosan (NCH) particles were synthesized by the ionotropic gelation method. Four milliliters of TPP solution was introduced drop-wise in a beaker glass containing 40 mL of CH solution under constant high-speed stirring at 10,000 rpm rate, room temperature, for 5 min using rotor-stator homogenizer (POLYTRON PT-3100D-Kinematica, Luzern, Swiss). The cross-linking reaction of TPP and Chitosan was then completed under ultrasonic irradiation for 20 min using an ultrasonic processor (Vibra Cell, Type 72434, 100 Watts, horn diameter: 1.0 mm, Fisher Scientific, Illkirch-Graffenstaden, France).

### 2.4. Preparation of Film Composites

The film composites of nanochitosan particles and cellulose nanocrystals (NCH-CNC) were manufactured by dispersing drop by drop 4 mL of CNC suspension in a beaker glass containing 40 mL of NCH suspension using a rotor-stator homogenizer at 5000 rpm, room temperature, for 5 min. The matrix NCH-CNC suspension was then irradiated using ultrasonic wave for 5 min, then followed by deaeration in a vacuum chamber for 15 min. The film composites were obtained by the solvent-casting method, in which the final NCH-CNC suspension was poured into circular type plastic petri dish with diameter 86.5 mm and height 12 mm and evaporated at room temperature and constant Relative Humidity (50% RH) for 2 days before peeling off of the dried film composites.

### 2.5. Density Measurement

The density of the liquids was measured by a Tensiometer (K-100-Krűs GmBH, Hamburg, Germany) composed of two parts: a holder (platinum-iridium) and an immersion body (silicon crystal). The measurement is based on the Archimedes principle with a range of 1 to 2200 kg/m^3^ and precision of ±3 kg/m^3^. The true (skeletal) density of samples was measured by gas pycnometer (AccuPyc 1330 from Micromeritics, GA, USA) using a 10 cm^3^ sample module and helium as filling gas (99.995% pure). Raw materials, which are chitosan and CNC, have densities greater than pure water, namely, 1456 kg/m³ for chitosan and 1576 kg/m³ for CNC.

### 2.6. Viscosity Measurement

The viscosity of liquid solutions was measured by rheometer Physica MCR 301 (Anton Par, GmbH, Graz, Austria) with torque (0.1–200) ± 0.001 μNm. Measuring type was used concentric cylinder chamber CC27/T200/Q1 (DIN 53019) at constant temperature 20 °C.

### 2.7. Diameter Particles Measurement

Nanoparticles diameters were measured by high-resolution Transmission Electron Microscopy, TEM (JEOL-2100F, JEOL Ltd., Tokyo, Japan) and Scanning Electron Microscopy, SEM (JEOL-2100F, JEOL Ltd., Tokyo, Japan). The sample was deposited on carbon-coated copper grids, and the negative staining was achieved using uranyLess solution (Delta Microscopies, Toulouse, France). The size and diameter distribution particle were measured by Image J (version 1.41 h) and origin pro-8 software (MA, USA, open source version). 

### 2.8. Water-Resistant

Water resistance of the films was evaluated by studying their behavior towards the water in the liquid and gas phase. Resistance to the liquid phase was imaged by a DSA-10 camera from Kruss GmbH, Hamburg, Germany. Liquid droplets were added on the surface of the film and then recorded. Image analysis was performed to calculate the volume decrease of the droplet through time

(1) $$Permeation\;rate=\frac{\Delta V}{\Delta t}$$

where Δ*V* was Droplet volume (μL) and Δ*t* was time (s). On the other hand, water vapour resistance was evaluated by covering a bottle containing a desiccant with the film. The bottle was then placed into a controlled chamber (HygroGen generator from Rotronic Instruments Ltd., Crawley, UK) with 75% relative humidity. The evolution of mass was recorded every day for one week. The equation below was used to calculate the water vapour permeability constant [29]

(2) $$K=\frac{\Delta m}{\Delta t}\left(\frac{\Delta y}{\Delta p}\right)A$$

where *K* is permeability coefficient (m^2^ s^−1^ Pa^−1^), Δ*m* is mass (kg), Δ*t* is time (s), Δ*y* is film thickness (m), Δ*p* is pressure difference (Pa), and *A* is Area (m^2^).

## 3. Results and Discussions

### 3.1. Nanochitosan Particles

**Figure 1:**
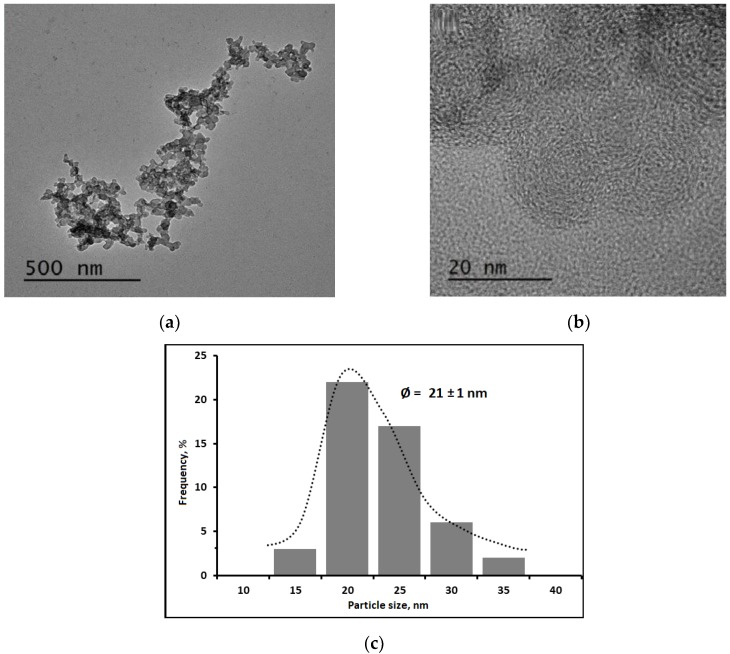
Transmission Electron Microscopy (TEM) images of Nanochitosan (NCH) particles: (**a**) 500 nm resolution, (**b**) 20 nm resolution, and (**c**) average particle size distribution.

Chitosan polymer solution was transformed into NCH suspension by adding TPP solution droplets through an ionotropic gelation mechanism. The TEM images are presented in Figure 1a,b. The images show that TPP is able to wrap the chitosan polymer chain. The cationic part of the chitosan polymer could then interact with the anionic part of TPP to form circular shape NCH particles with an average particle size distribution is 21 ± 1 nm (Figure 1c).

### 3.2. Liquid Properties

The nanofluids consist of two main constituents, namely, a continuous phase that is an acetic acid solution and a dispersed phase consist of NCH and CNC. The interactions between continuous and dispersed phases determine the behavior of the final solution in static and dynamic conditions. The static condition was dominated by gravitational force, whereas dynamic condition was dominated by continuous phase motion. When continuous phase moved, its velocity induced a momentum transfer to the discrete phase, which caused them to move either with the same or a different velocity. The inertia of the discrete phase determines what kind of response is going to be generated. NCH and CNC were mixed together to form a composite in the liquid phase. The density and the viscosity of liquid suspensions were then determined. These parameters determine the microscopic activity of the liquid solution that is crucial to optimize the process and the key properties of the NCH-CNC composite film.

#### 3.2.1. Liquid Density

0.1 M acetic acid solutions and CNC suspension have a density comparable to that of pure water, namely, 1000 kg/m^3^. All liquid samples, whether CH, NCH, or both, also have a very close density to that of pure water. Density variations were very small and did not exceed ±1 kg/m^3^, that is, below the measurement precision of density determination.

#### 3.2.2. Liquid Viscosity

**Figure 2:**
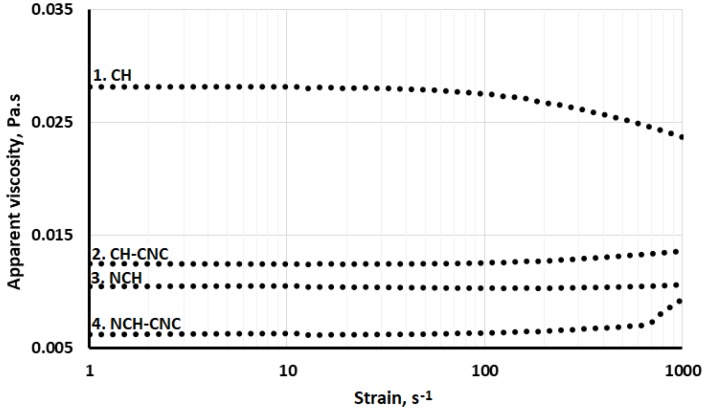
Liquids behavior under different shear rates.

Acetic acid and CNC suspension had a viscosity of 1 mPa s, which is the same as pure water. Consequently, any change in the apparent viscosity arises from the suspension of particles. Figure 2 shows the variation of the apparent viscosity as a function of the shear stress for different solutions and suspension used in this study. The CH shows that at low shear rates (the limit of zero shear rate), the apparent viscosity is almost constant. The subsequent Newtonian viscosity (η) is close to 28 mPa s. However, its slope gradually decreases with the strain rate above 100 s^−1^, which represents the transition from Newtonian to the non-Newtonian regime. According to literature, this behavior could be explained by the propensity of polymer molecules to stretch under the effect of the shear rate [30]. The entanglement–disentanglement extent of chitosan depends on the shear rate, which is characterized by the changing slope of the apparent viscosity [31]. Similar trends have been reported in the literature for varying concentrations of acetic acid, including the one used in the present study [32,33].

NCH suspension is commonly obtained by an added droplets of the TPP solution followed by a mixing step [34,35]. TPP influences mainly the two-fluid characteristics, namely, cohesive force and selfdeformation of the particle inside. The cohesive force of NCH is smaller than the CH leading to a decrease of the apparent viscosity. The NCH shows a Newtonian fluid behavior within the range of shear rates used in this study. This signifies that NCH particles are able to maintain their shape under the applied shear rate range. These results were confirmed by TEM micrographs (Figure 1), which showed that NCH has a spherical shape.

Generally, the mixing of polymers allows for the improvement of the properties of composite materials [36]. This is also the expected goal of mixing NCH and CNC particles in the present study. Indeed, CNC modifies the flow behavior of the solution. CNC has the ability to the selfarrangeme in solution because of its zeta potential [37,38]. This affects the behavior of the nanofluid mixture in static and dynamic conditions. In dynamic conditions, CNC compensates for the flow of continuous phase by translational motion, which tries to minimize the drag force. The CNC particles reoriented themselves to be in line with the flow direction. During this process, the stress over CNC surface changes and simultaneously influences the velocity field of the fluid medium around CNC.

**Figure 3:**
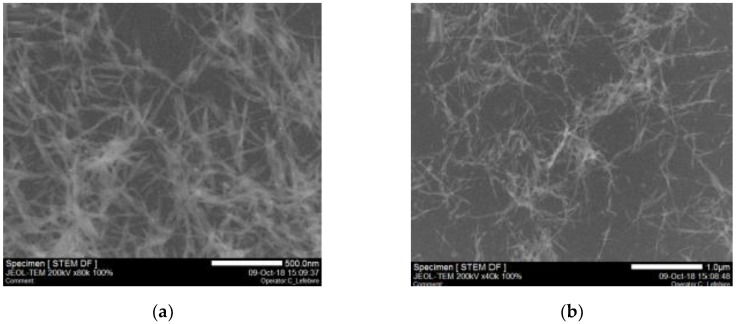
STEM micrographs of Cellulose Nanocrystals (CNC) particle; (**a**) 500 nm resolution and (**b**) 1 μm resolution.

Under shear rate flow, it seems that the flow behavior is dominated by CNC rather than NCH. This could be explained by the difference of particle motion under shear rate according to their shape. The Scanning Transmission Electron Microscopy (STEM) micrograph below shows CNC shape and morphology. CNC is the crystalline part of cellulose that has a rod-shaped geometry, which can be considered as spheroidal shape (Figure 3).

In order to take account of the distribution of orientation, a nondimensional parameter, namely, Peclet number for shear flow case, needs to be considered

(3) $$P_{e}=\frac{\dot{\gamma }}{D_{r}}$$

where $\dot{\gamma }$ is shear rate and $D_{r}$ is time scales for Brownian motion. The Peclet number (*P_e_*) represents a comparison between hydrodynamic and Brownian motion [39]. CNC’s shape could be approximated as elongated with an aspect ratio much higher than unity. The rotational Brownian diffusion coefficient (*D_r_*) can be obtained by the equation

(4) $$D_{r}=\frac{3kT\left(ln2r_{p}-1/2\right)}{8\pi \eta_{s}a^{3}}$$

where *k* is Boltzmann constant (m^2^ kg s^−2^ K^−1^), *T* is Absolute temperature (K), *r_p_* is axis ratio, *η_s_* is viscosity (Pa s), and *a* is axis of the symmetry (m). NCH nanoparticles have a quite spherical shape. Their rotational Brownian diffusion coefficient can be obtained by the equation

(5) $$D_{r}=\frac{3kT}{8\pi \eta_{s}a^{3}}$$

**Figure 4:**
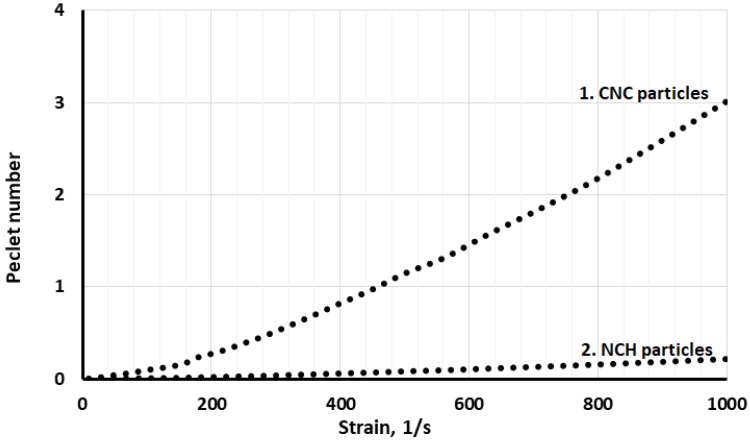
Peclet number of CNC and Nanochitosan (NCH) particles.

As can be seen from Figure 4, the Peclet number of CNC is much higher (about 15-fold) than NCH particles, which means that CNC is the most dominant component under dynamic conditions.

### 3.3. Hydrodynamic Behavior

Hydrodynamic diameter observed by Dynamic light scattering (DLS) supports the TEM micrographs result, which shows that TPP squeezes the diameter of chitosan chain polymer and form circular shape particles. NCH in suspension has a much higher hydrodynamic diameter than the crystalline diameter observed in TEM, which implies a degree of aggregation of the nanoparticles while remaining stable for several hours in suspension. NCH suspensions have comparable hydrodynamic diameter when adding varying CNC concentrations are presented in Table 1.

NCH and CNC are both charged particles characterized by the surface (zeta) potential. The electric potential over the surface of the particles is the cause of their electrophoretic mobility [40,41,42]. Zeta-potential located at the shear plane of the particles describes how important those effects are. Higher potential represents denser surface energy. NCH and CNC have positive and negative charges, respectively. When they are mixed together, their interaction leads to a slightly different potential. Note that the zeta potential is also related to the morphology of particles. 30 NCH and CNC have different shapes (see Figure 1 and Figure 3) that affect their behavior differently when dynamic motion is applied to the suspension.

### 3.4. Film Morphology

**Figure 5:**
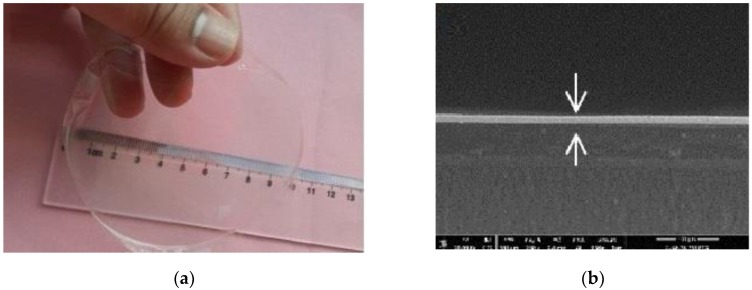
(**a**) Photographic image of film composite NCH-CNC and (**b**) film thickness measurement.

The evaporation process occurs at the interface between the liquid solution and air, and is mainly driven through diffusivity. This process takes place at the interface caused by the difference of partial pressures between gas and liquid phases [43]. Various mechanisms are involved when the liquid in the bulk region moves to the interface, but the main hydrodynamic flow mechanism depends on the osmosis pressure gradient [44]. Figure 5 presents the NCH-CNC film and SEM micrographs (side view as thickness). The evaporation of the nanofluid reduces the intermolecular distances and form a continuous film. Smaller distance allows the attractive forces to overcome the repulsive forces. The unbalanced force leads to the coalescence between molecules in order to minimize the surface energy. The whole activity during evaporation time determines the final film thickness [44,45,46]. Chitosan has good film formation ability, but CNC particle is crystalline and the coalescence between particles does not occur [46].

**Figure 6:**
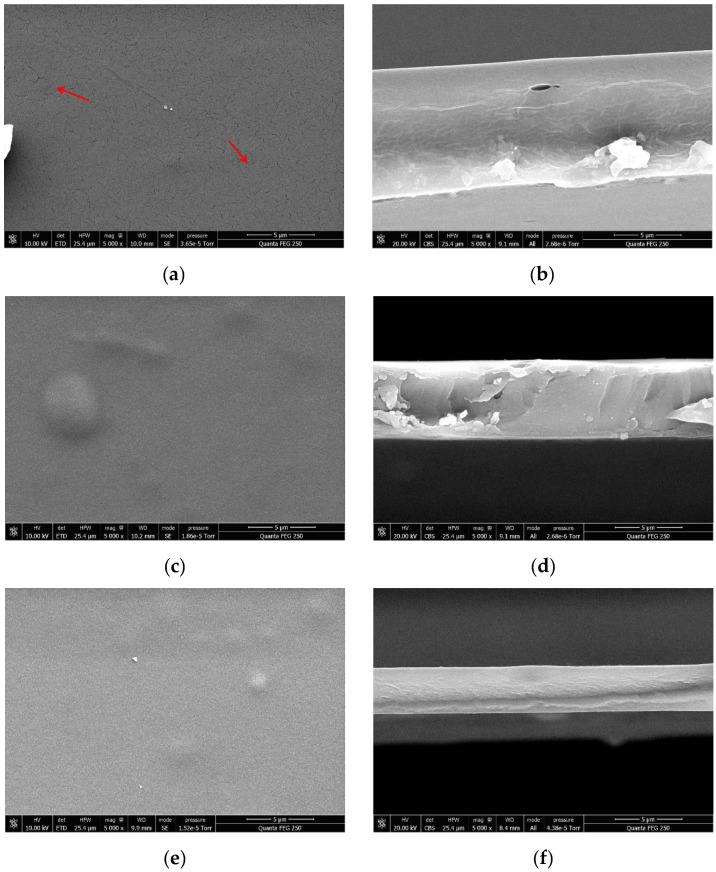
SEM micrographs with 5000x magnification of film composites: (**a**) chitosan (CH) top view, (**b**) CH Side view, (**c**) CH-CNC top view, (**d**) CH-CNC side view, (**e**) NCH-CNC top view, and (**f**) NCH-CNC side view.

Figure 6 presents the CH, CH-CNC, and NCH-CNC films, and shows that the morphology of films is similar. Nevertheless, the surface of the chitosan film contains scratch at micrometer scale that may be due to some discontinuities formed during the film formation process. On the contrary, NCH-CNC composites are able to form a smooth and continuous film. It should be noted that close up of the surface did not permit one to show any nanoobjects, and the surface appeared completely smooth and homogeneous.

**Figure 7:**
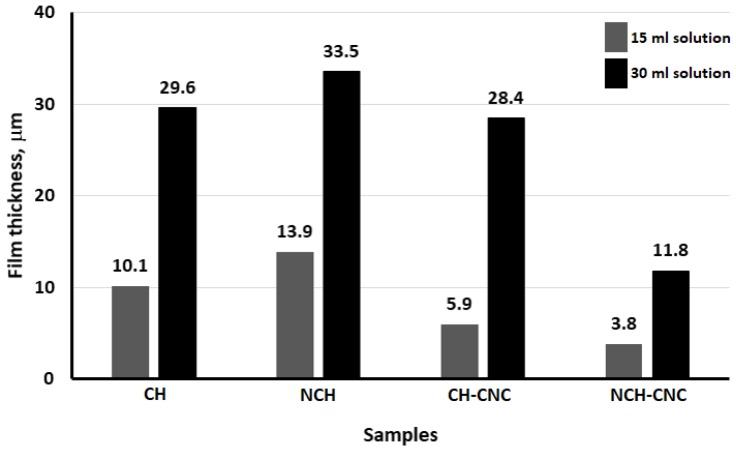
Film thickness measurement.

We speculate that these results are the reason behind reported works that found that CNC is able to increase the tensile strength and other mechanical properties of composite films [47,48]. Indeed, CNC molecules are able to rearrange themselves and make a network that tries to minimize their electrostatic interactions, which leads to a good distribution of the discrete phase [49]. Therefore, CNC contributes to maintaining a certain distance between each particle and tries to establish the equilibrium potential. When a liquid suspension of CH-CNC nanocomposite is evaporated, this equilibrium potential tends to overcome the instability during the film formation process. The lack of that potential equilibrium is the main reason that film formation ceases in the absence of CNC. Figure 7 displays the film thickness of four different samples at the same volume solution. 

### 3.5. Statistical Analysis

**Figure 8:**
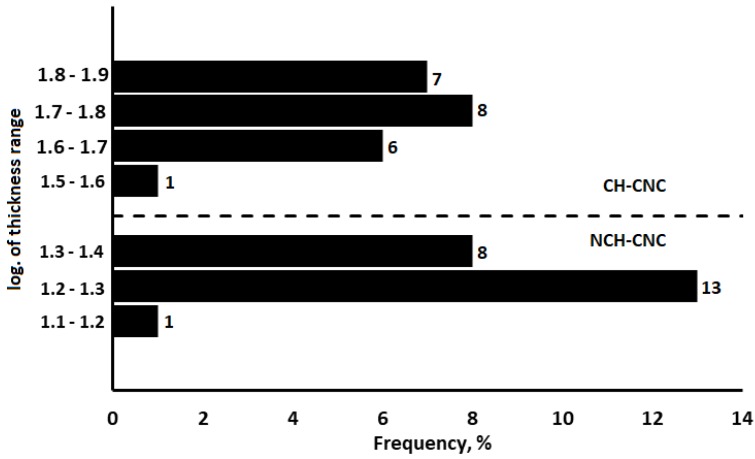
T-test graph of CH-CNC and NCH-CNC film thickness.

In order to determine if the CNC has a real effect on the thickness of the nanocomposite film, a t-test analysis was performed. The results of the test, shown in Figure 8, indicate a statistically significant difference in the variance for CH-CNC, compared to NCH-CNC. Thus, we can be assured that CNC will make the film network more rigorous.

### 3.6. Water-Resistance

Chitosan-based films have low water resistance, but the addition of CNC is one of the most promising ways to overcome this problem. In the presence of the CNC, the water-resistance properties (vapour or liquid) of CH and NCH are modified is presented in Table 2.

**Figure 9:**
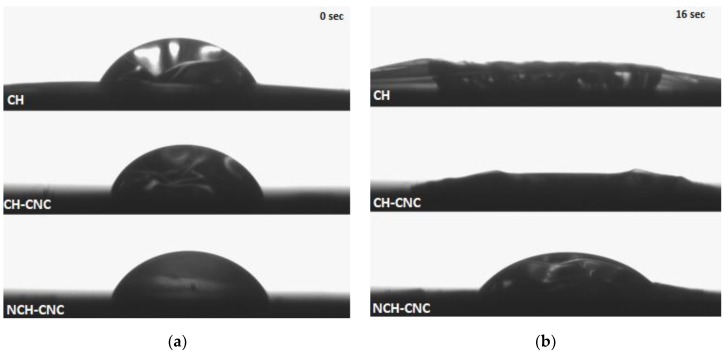
Record of water droplet penetration on film composites: (**a**) initial condition at 0 s, (**b**) after film absorb water liquid at 16 s.

This reinforcing effect is more significant for resistance to liquid water. Figure 9 shows the behavior of the film with nanocrystalline cellulose towards a water droplet. Thus, only NCH-CNC could retain water droplets more than five minutes where other samples absorbed water and were damaged in less than twenty seconds. The figures below show how the water penetrates when the water liquid drops on the surface of the film. It appears that water spread randomly to all directions in the pure chitosan film. CNC addition leads to a more circular shape of water penetration. Under gravity only, the water moves to all directions with the same force. The tendency to form more circular shapes of the film show that water resistance force spread more homogeneously when CNC was involved.

## 4. Conclusions

We produced chitosan solution and nanochitosan suspension added with nanocellulose and evaluated their properties: zeta potential and hydrodynamic diameter of the colloidal suspension as well as density and viscosity of the solution and suspension. Differences in properties in the liquid phase are clearly visible when one of the constituents is in the nanoform. We then produced films from the evaporation of the different solutions and suspensions and evaluated their properties as a function of their constitution. These were clearly shown to be dependent on the nature of the chitosan constituent (polymeric or nanoparticle) and the presence of nanocellulose. It was found that film morphology was smoother when the constituent was in nanoform and that film thickness was decreased by the adjunction of nanocellulose. Finally, it was shown that nanocellulose was essential for improving the water resistance (in liquid or vapour form) of the films.

## Figures and Tables

**Table 1:** Digital Light Scattering (DLS) measurement.

Sample	Hydrodynamic Diameter (nm)	PDI	Zeta Potential (mV)
NCH	357.1	0.522	+62.0
NCH + CNC	377.6	0.453	+59.3

**Table 2:** Water-resistance properties of film composites.

Sample	Water liquid Penetration Rate (10^−3^ μL s^−1^)	Water Vapour Permeability Constant (10^−15^ m^2^ s^−1^ Pa^−1^)
CH	8.3	3.39
NCH	0.3	4.65
CH-CNC	0.5	1.98
NCH-CNC	0.1	1.25
